# Supernumerary Kidney With Horseshoe Fusion Anomaly: A Case Report and Comprehensive Review of the Literature

**DOI:** 10.7759/cureus.111217

**Published:** 2026-06-20

**Authors:** Perarivalan Ilango, Arun SV, S Rohith, Keerthana Suresh, Rajeswaran R, Venkata Sai

**Affiliations:** 1 Radiodiagnosis, Sri Ramachandra Institute of Higher Education and Research, Chennai, IND; 2 Diagnostic Radiology, Sri Ramachandra Institute of Higher Education and Research, Chennai, IND; 3 Radiology, Sri Ramachandra Institute of Higher Education and Research, Chennai, IND

**Keywords:** congenital renal anomaly, ct urography, duplex collecting system, horseshoe kidney, renal fusion anomaly, supernumerary kidney

## Abstract

A supernumerary kidney is an extremely rare congenital anomaly of the genitourinary tract characterized by the presence of an accessory kidney with a separate capsule, vascular supply, and collecting system. It may be an isolated anomaly or associated with other congenital anomalies of the genitourinary tract. The fusion of a supernumerary kidney in a horseshoe configuration is a very rare developmental anomaly with few cases reported in the literature. Clinical presentation varies from incidental finding to abdominal pain, urinary tract infection, hydronephrosis, nephrolithiasis, or other complications. We report the case of a 26-year-old man in whom multimodality imaging was performed for renal evaluation in view of increased frequency of micturition. Initial ultrasonography and Doppler evaluation demonstrated anomalous renal morphology with preserved corticomedullary differentiation and no evidence of hemodynamically significant renal artery stenosis. Cross-sectional imaging further delineated the abnormal renal anatomy, confirming the presence of an accessory renal moiety with a fusion anomaly. Renal function and urinary drainage were preserved. No evidence of hydronephrosis or obstructive changes were seen. This case highlights the importance of multimodality imaging in characterizing rare congenital renal anomalies, differentiating a supernumerary kidney from more common anomalies such as duplex kidney or horseshoe kidney, and facilitating accurate diagnosis for clinical management. We also review the embryological basis, imaging characteristics, associated anomalies, and previously reported literature.

## Introduction

Congenital anomalies of the kidneys and urinary tract (CAKUT) include a wide range of developmental malformations involving renal number, position, fusion, rotation, and collecting system abnormalities. One of the rarest congenital renal anomalies is the supernumerary kidney, which is characterized by an accessory renal unit that has its own capsule, blood supply, and collecting system, and is therefore distinct from the more common duplex kidney or duplicated collecting system [1,2].

The exact incidence of supernumerary kidney is unknown due to its extreme rarity, with fewer than 100 cases reported in the literature. The anomaly is believed to result from abnormal division of the nephrogenic cord into two metanephric blastemas, often associated with partial or complete duplication of the ureteric bud in early embryogenesis [1,3]. The supernumerary kidney may be completely separate or partially fused with the ipsilateral native kidney, depending upon the degree of developmental separation and fusion.

Supernumerary kidneys are usually smaller than the normal kidney and are located caudal to the ipsilateral renal unit, but cranial and ectopic locations have been reported [2,4]. The drainage pattern of the ureters can be an independent insertion into the bladder or a fusion with the collecting system of the ipsilateral kidney. Associated anomalies include horseshoe kidney, ureteral atresia, vaginal atresia, duplicated urethra, ventricular septal defects, meningomyelocele, coarctation of the aorta, and cloacal abnormalities [3,5].

The clinical presentation is extremely varied. Many cases are incidentally seen on imaging performed for other indications. Symptomatic patients may present with abdominal pain, flank pain, recurrent urinary tract infections, hydronephrosis, nephrolithiasis, pyonephrosis, hypertension, or rarely neoplastic change, including Wilms tumor and renal cell carcinoma [2,6].

The main difficulty in diagnosis is distinguishing a supernumerary kidney from other congenital anomalies such as a duplex kidney, crossed fused ectopia, and a horseshoe kidney. Duplex kidneys, although far more common, are a duplication of the pelvicalyceal system within a single renal parenchymal unit, not the formation of a true accessory organ. Accurate diagnosis is therefore dependent on cross-sectional imaging, especially computed tomography (CT) urography or magnetic resonance urography, which can accurately delineate renal parenchymal anatomy, vascular supply, and collecting system configuration [1,4].

Supernumerary kidney associated with horseshoe fusion morphology is exceptionally rare, and only isolated case reports have been described in the literature [5,7]. Its clinical identification is important not only to avoid diagnostic misclassification but also because the anatomic complexity may influence surveillance strategies, management decisions, and planning of future surgeries or interventions.

We present an unusual case of a supernumerary kidney with a horseshoe fusion anomaly in a young male adult, highlighting the diagnostic importance of multimodality imaging and discussing the embryological and radiological characteristics of this uncommon congenital entity.

## Case presentation

A 26-year-old male presented with increased frequency of micturition and underwent radiological evaluation. There was no prior history of renal surgery, congenital genitourinary malformations, recurrent urinary tract infection, nephrolithiasis, or significant systemic illness. Clinical examination was unremarkable, and there were no features suggestive of urinary tract obstruction or renal dysfunction.

Initial ultrasonography demonstrated anomalous renal morphology with preserved corticomedullary differentiation. The right kidney measured approximately 11.8 × 4.0 cm, and the left kidney measured 9.4 × 3.6 cm. Both kidneys demonstrated normal echotexture without focal parenchymal abnormality. An additional reniform parenchymal structure was identified inferior to the native right kidney, raising suspicion for a supernumerary renal moiety (Figure 1).

**Figure 1:**
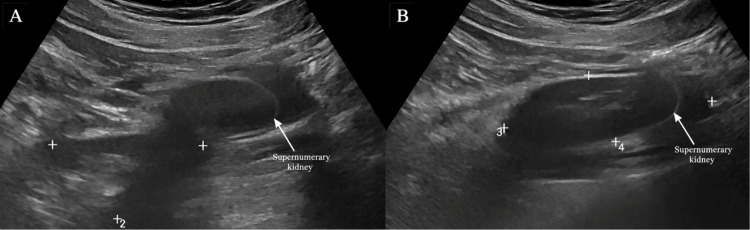
(A) Grayscale ultrasonographic image demonstrating an additional well-defined renal parenchymal structure adjacent to the native kidney, suggestive of a supernumerary kidney (arrow). (B) Grayscale ultrasonographic image in a different plane demonstrating the supernumerary kidney as a distinct accessory renal moiety with preserved corticomedullary echotexture (arrow).

Subsequent color Doppler evaluation demonstrated preserved intrarenal vascularity with normal arterial waveforms. The right hilar artery demonstrated a peak systolic velocity (PSV) of 106 cm/s and a resistive index (RI) of 0.6. Segmental arterial branches within the right kidney demonstrated PSV values ranging from 35 to 61 cm/s with RI values between 0.5 and 0.6. The left hilar artery demonstrated a PSV of 74 cm/s with an RI of 0.6. Vascularity was also demonstrated within the anomalous connecting renal parenchymal tissue, which showed a PSV of approximately 68.5 cm/s, end-diastolic velocity (EDV) of 28 cm/s, RI of 0.6, and acceleration time of 0.06 seconds. No Doppler evidence of hemodynamically significant renal artery stenosis was identified (Figure 2).

**Figure 2:**
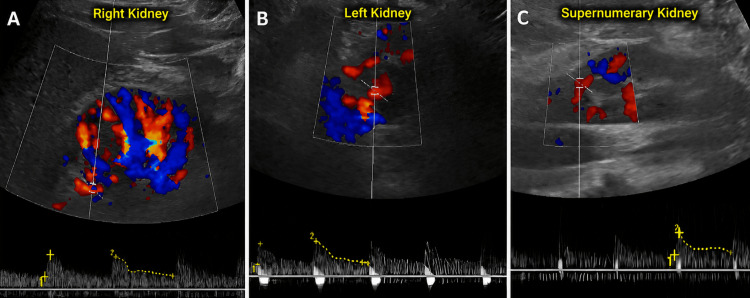
(A) Color and spectral Doppler evaluation of the right native kidney demonstrates preserved intrarenal vascularity with normal arterial waveforms. (B) Color and spectral Doppler evaluation of the left native kidney demonstrates preserved perfusion with maintained arterial flow signals. (C) Color and spectral Doppler evaluation of the supernumerary kidney demonstrates an identifiable vascular supply with preserved perfusion and arterial flow waveform, confirming a vascularized, functioning renal parenchyma.

Given the unusual sonographic findings and the need for detailed anatomical characterization, contrast-enhanced CT urography with delayed excretory phase imaging was performed.

CT urography demonstrated a complex congenital renal anomaly consisting of three renal moieties: a native right kidney, a supernumerary right renal moiety located inferior to the native right kidney, and a native left kidney. The native right kidney measured approximately 10.9 × 4.6 cm and demonstrated normal enhancement without focal parenchymal lesions. The left kidney measured approximately 8.7 × 3.6 cm and was relatively smaller in size but demonstrated preserved enhancement and corticomedullary differentiation. The supernumerary right renal moiety showed preserved cortical enhancement, maintained corticomedullary differentiation, and a distinct reniform morphology, consistent with functioning renal parenchyma (Figure 3).

**Figure 3:**
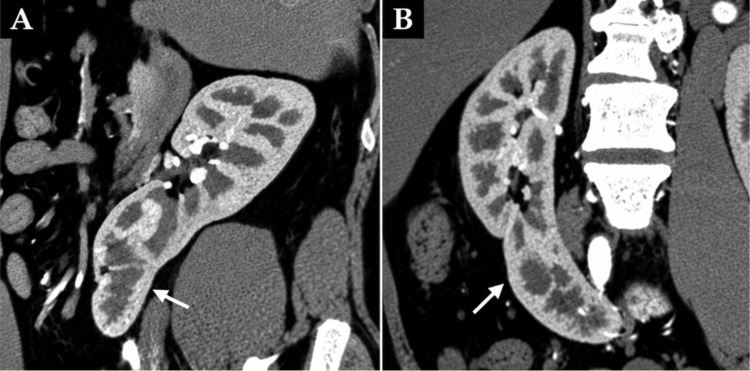
(A) Coronal contrast-enhanced CT urographic image demonstrating an accessory renal moiety inferior to the native kidney, showing preserved parenchymal enhancement consistent with a supernumerary kidney (arrow). (B) Coronal contrast-enhanced CT urographic image in a different plane demonstrating the supernumerary kidney as a distinct accessory renal moiety with preserved corticomedullary differentiation (arrow). CT - Computed Tomography

Both native kidneys demonstrated features suggestive of partial duplication of the collecting system. Delayed excretory phase images demonstrated lower pole fusion through a functioning parenchymal isthmus traversing the midline at the L3-L4 vertebral level anterior to the great vessels, consistent with horseshoe fusion morphology (Figure 4).

**Figure 4:**
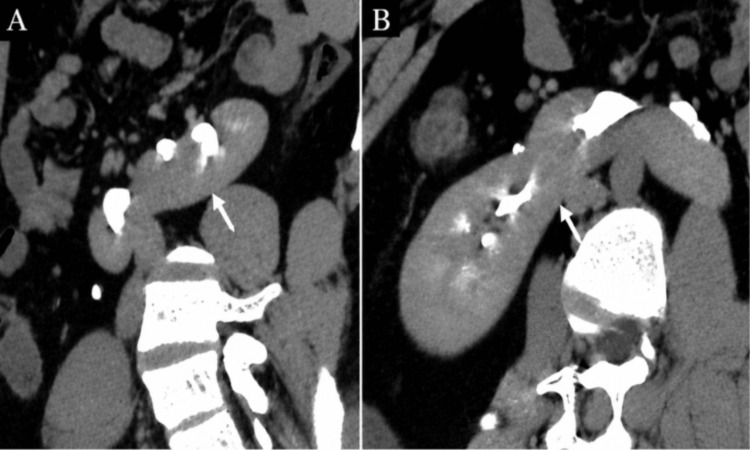
(A) Coronal contrast-enhanced CT urographic image demonstrating lower pole fusion of the renal units by a parenchymal isthmus traversing the midline anterior to the vertebral column, consistent with horseshoe fusion morphology (arrow). (B) Coronal contrast-enhanced CT urographic image in a different plane demonstrating the renal parenchymal isthmus connecting the fused lower poles anterior to the aorta and vertebral column, consistent with horseshoe fusion morphology (arrow). CT - Computed Tomography

Arterial-phase reconstructed images demonstrated an independent vascular supply to the supernumerary renal moiety through a separate arterial branch arising directly from the abdominal aorta. Independent venous drainage into the inferior vena cava was also identified. These findings supported the diagnosis of a true supernumerary kidney rather than a duplicated collecting system.

Excretory phase imaging demonstrated satisfactory contrast excretion and preserved urinary drainage without hydronephrosis, ureteric obstruction, nephrolithiasis, intraluminal filling defects, or urinary bladder abnormality. The collecting system of the supernumerary right renal moiety appeared to communicate with the left-sided collecting system, with apparent convergence of the ureteric drainage pathways. In addition, the left collecting system demonstrated partial duplication, with upper and lower pole moieties appearing to unite at approximately the L2-L3 vertebral level before distal continuation as a common ureter. No ectopic ureteric insertion, ureterocele, or vesicoureteric abnormality was identified (Figure 5).

**Figure 5:**
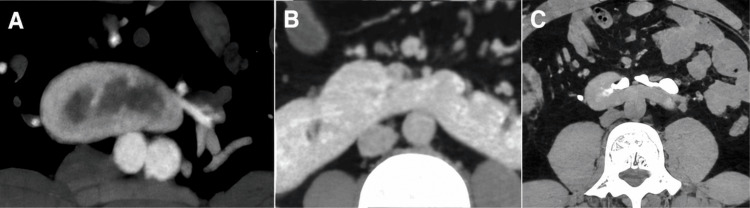
Additional CT urogram images demonstrating the vascular and ureteric anatomy of the supernumerary renal moiety. (A) Axial contrast-enhanced CT arterial phase image demonstrating an independent arterial branch arising directly from the abdominal aorta supplying the supernumerary right renal moiety. (B) Axial contrast-enhanced CT venous phase image demonstrating separate venous drainage of the supernumerary renal moiety into the inferior vena cava. (C) Delayed excretory phase CT urogram image demonstrating the ureter arising from the supernumerary right renal moiety, coursing medially and appearing to communicate with the left-sided ureteric drainage pathway. CT – Computed Tomography; IVC – Inferior Vena Cava.

Based on the multimodality imaging findings, a diagnosis of a supernumerary right kidney with a horseshoe fusion anomaly, associated bilateral partial duplication of the collecting systems, and complex ureteric drainage anatomy was established.

In the absence of obstructive uropathy, infection, calculous disease, impaired drainage, or functional compromise, conservative management and periodic clinical follow-up with blood pressure monitoring and renal function assessment were advised.

## Discussion

A supernumerary kidney is characterized by the presence of an accessory renal organ with its own capsule, separate vascular supply, and independent collecting system [1,2]. This entity is to be distinguished from the more common duplex kidney, in which there is duplication of the collecting system within a single renal parenchymal unit, not the development of a separate accessory renal moiety [1]. A supernumerary kidney is a rare entity with variable anatomy and continues to be a diagnostic dilemma, and requires cross-sectional imaging for definitive characterization.

The embryological cause of the anomaly is thought to be an abnormal division of the nephrogenic cord into two metanephric blastemas in the fifth to seventh weeks of gestation with partial or complete duplication of the ureteric bud [2,3]. This developmental error results in the formation of an accessory renal unit that may be entirely separate or partially fused with the ipsilateral native kidney. The fusion depends on the time of separation in embryology and the subsequent ascent of the kidney. Abnormal fusion may rarely result in complex configurations such as the horseshoe morphology observed in the present case [4,5].

Supernumerary kidneys are mostly smaller than the native kidneys and are usually located caudal to the ipsilateral renal unit, but cranial, pelvic, and ectopic locations have also been reported [2,6]. Drainage patterns are variable; the accessory kidney may possess an independent ureter draining separately into the urinary bladder or may share a partially fused collecting system with the ipsilateral kidney. This anatomical heterogeneity contributes significantly to diagnostic difficulty, particularly on ultrasound, where complex fusion anomalies may be misinterpreted as duplex kidneys or conventional horseshoe kidneys.

A supernumerary kidney has a variable clinical presentation. Many are asymptomatic and are discovered serendipitously on imaging done for other reasons [2]. Patients presenting with symptoms may have abdominal pain, flank pain, recurrent urinary tract infections, hydronephrosis, pyelonephritis, nephrolithiasis, hematuria, or hypertension [3,6]. Rare complications like pyonephrosis, renal dysfunction, and malignant transformation, such as Wilms tumor, clear cell carcinoma, or urothelial malignancy, have also been reported [2,7]. In the present case, the absence of obstruction, infection, calculi, or impaired drainage favored conservative management.

Differential diagnosis from other congenital renal anomalies was the main consideration in this case. A duplex kidney remains the most important differential diagnosis, as sonically duplicated collecting systems may simulate accessory renal tissue [1]. A true duplex kidney is a single encapsulated renal unit with duplicated pelvicalyceal systems, whereas a supernumerary kidney is a separate organ with independent vascular and collecting anatomy. Another important differential diagnosis is horseshoe kidney, a relatively common fusion anomaly in which the lower poles of the native kidneys are fused and ascent is usually arrested by the inferior mesenteric artery [3]. Crossed fused renal ectopia may resemble complex renal fusion anomalies but distinguishes itself by abnormal migration and ureteric course.

Imaging plays a pivotal role in establishing the diagnosis. Ultrasonography is usually the first imaging modality because it is available and does not expose the patient to radiation, but its diagnostic accuracy can be limited in complex congenital anomalies due to limited anatomical delineation [6]. Additional vascular assessment is provided by Doppler ultrasound but this remains insufficient to completely define anomalous renal fusion anatomy. CT urography provides excellent anatomical resolution for detailed evaluation of renal morphology, vascular supply, collecting system anatomy, and associated complications [1,4]. Magnetic resonance urography can be an alternative in younger patients or those who want to avoid radiation.

The rarity of a supernumerary kidney with a horseshoe fusion anomaly makes literature comparison limited. Literature describes isolated cases of triple kidneys, supernumerary kidneys with partial fusion, and very rare horseshoe variants [4,5]. The Indian Journal of Nephrology case describing a supernumerary kidney with a third renal pole and horseshoe component demonstrated anatomical similarities to the present case, underscoring the embryological plausibility of such complex fusion anomalies [4]. Likewise, a previously published Cureus case describing a triple kidney with horseshoe malformation emphasized the importance of CT urography in differentiating true supernumerary anatomy from duplicated systems [5]. The present case further stresses the importance of multimodality imaging in detecting atypical congenital renal configurations and avoiding diagnostic misclassification, when compared to these reports.

Recognition of this anomaly has important clinical implications. Misdiagnosis may lead to inaccurate surgical planning, incomplete evaluation of vascular anatomy, or inappropriate intervention. Even in asymptomatic patients, accurate documentation is valuable for long-term surveillance and future urological or abdominal procedures, where anomalous vascular supply and collecting system anatomy may significantly alter operative risk.

## Conclusions

A supernumerary kidney with horseshoe fusion morphology is an exceptionally rare congenital renal anomaly. In the present case, the diagnosis was established by multimodality imaging demonstrating a supernumerary right renal moiety located inferior to the native right kidney, fusion of the accessory renal moiety with the left kidney through a functioning parenchymal isthmus, independent arterial supply arising from the abdominal aorta, separate venous drainage into the inferior vena cava, and complex collecting system anatomy with associated partial duplication of the native collecting systems. Delayed excretory phase imaging further demonstrated preserved urinary drainage without evidence of obstruction or functional compromise.

This case highlights the importance of CT urography in accurately delineating renal parenchymal anatomy, vascular supply, fusion morphology, and ureteric drainage patterns, thereby allowing confident differentiation from duplex kidney, crossed fused ectopia, and conventional horseshoe kidney. Recognition of such rare anatomical variants is important for accurate diagnosis, surgical planning, and avoidance of inadvertent vascular or ureteric injury during future abdominal, vascular, or urological interventions. Given the rarity of this constellation of findings, documentation of such cases contributes to the existing literature and enhances awareness among radiologists, urologists, and clinicians regarding the spectrum of complex congenital renal fusion anomalies.
